# Keratitis caused by the recently described new species *Aspergillus brasiliensis*: two case reports

**DOI:** 10.1186/1752-1947-4-68

**Published:** 2010-02-24

**Authors:** Palanisamy Manikandan, János Varga, Sándor Kocsubé, Rajaraman Revathi, Raghavan Anita, Ilona Dóczi, Tibor Mihály Németh, Venkatapathy Narendran, Csaba Vágvölgyi, Madhavan Bhaskar, Chockaiya Manoharan, Robert A Samson, László Kredics

**Affiliations:** 1 Aravind Eye Hospital and Postgraduate Institute of Ophthalmology, Avinashi road, Coimbatore 641 014, Tamil Nadu, India; 2 CBS Fungal Biodiversity Centre, Uppsalalaan 8, 3584 CT Utrecht, The Netherlands; 3 Department of Microbiology, Faculty of Science and Informatics, University of Szeged, Közép fasor 52, H-6726 Szeged, Hungary; 4 Department of Clinical Microbiology and Diagnostics, Faculty of Medicine, University of Szeged, Somogyi Béla tér 1, H-6725 Szeged, Hungary; 5 Department of Botany & Microbiology, AVVM Sri Pushpam College, Poondi 613503, Tanjavur, India; 6 Department of Microbiology, Coimbatore Medical College, Coimbatore 641 014, Tamil Nadu, India

## Abstract

**Introduction:**

Human infections caused by *Aspergillus brasiliensis *have not yet been reported. We describe the first two known cases of fungal keratitis caused by *Aspergillus brasiliensis*.

**Case presentations:**

A 49-year-old Indian Tamil woman agricultural worker came with pain and defective vision in the right eye for one month. Meanwhile, a 35-year-old Indian Tamil woman presented with a history of a corneal ulcer involving the left eye for 15 days. The fungal strains isolated from these two cases were originally suspected to belong to *Aspergillus *section *Nigri *based on macro- and micromorphological characteristics. Molecular identification revealed that both isolates represent *A. brasiliensis*.

**Conclusion:**

The two *A. brasiliensis *strains examined in this study were part of six keratitis isolates from *Aspergillus *section *Nigri*, suggesting that this recently described species may be responsible for a significant proportion of corneal infections caused by black Aspergilli. The presented cases also indicate that significant differences may occur between the severities of keratitis caused by individual isolates of *A. brasiliensis*.

## Introduction

Certain *Aspergillus *species, mainly *A. flavus*, *A. terreus*, *A. fumigatus *and *A. niger *have long been regarded as important pathogens in eye infections, especially keratitis [1]. Other members of the genus less frequently occurring in keratitis include *A. glaucus*, *A. ochraceus *and *A. tamarii *[1,2]. The identification at the species level of *Aspergillus *strains causing keratomycosis would be of great importance since the pathogenic potential and antifungal susceptibilities may substantially vary between different species of the genus. Herein we report the first two known cases of fungal keratitis caused by the recently described species *A. brasiliensis*.

## Case presentations

A 49-year-old, Indian Tamil woman agricultural worker came with pain and defective vision in the right eye for one month. The symptoms started after she was exposed to paddy husk. At the time of presentation she was using 5% topical natamycin and gatifloxacin eye drops prescribed by her ophthalmologist. She had no significant past ophthalmic history or medical history. On examination, the visual acuity in her right eye was 5/60. Slit lamp evaluation of the right eye revealed a full thickness corneal abscess involving the nasal 1/3^rd ^of the cornea and the adjacent limbus with a localized thick exudation extending from the endothelial side on to the iris, partly covering the pupillary area. Routine microbiological workup did not reveal any organism in smear studies, but a black *Aspergillus *was identified from culture after four days (designated as strain 832/06). Based on clinical impression, topical itraconazole and 200 mg oral ketoconazole twice a day were added to natamycin, but the ulcer perforated by the fourth day. Topical natamycin was replaced by 0.15% amphotericin B and a therapeutic corneal transplantation was performed. Part of the iris, which was covered by the exudation, was found to be necrotic and was excised. The anterior chamber was washed with 80 μg/ml amphotericin B. Topical amphotericin B, clotrimazole and oral ketoconazole were continued post-operatively with topical ketorolac and 2% cyclosporine A drops. The graft remained clear initially but with severe fibrinous reaction in aqueous. On 12 days, the infection seemed to be eradicated but extensive peripheral anterior synechiae and post synechiae formed and a mature cataract developed. Ultrasonic B scan showed a clear vitreous. Cataract aspiration and synechiolysis were done after 14 days. Topical prednisolone acetate suspension was also started post-operatively.

By the 40^th ^postoperative day, however, peripheral synechiae reappeared at the inferior 2/3^rd ^circumference and the intraocular pressure (IOP) started to rise (the IOP spike was 46 mmHg). The glaucoma was controlled medically with 0.5% timolol and 2% Pilocarpine drops. Six months later, a penetrating optical graft was performed with synechiolysis. Though the graft remained clear for three months, the IOP started to rise with topical steroids, which needed enhanced medical therapy with Alphagan. However, the recalcitrant glaucoma necessitated a cyclodestructive procedure with Diode laser. Though the intraocular pressure was controlled, the graft failed to recover.

A 35-year-old Indian Tamil woman presented with a history of corneal ulcer involving the left eye for the past 15 days. She had been treated with 5% natamycin by her local ophthalmologist. She also gave a history of enucleation of the right eye following trauma sustained a year earlier. On examination, the vision in the left eye was 2/60. Slit lamp evaluation revealed a mild, central corneal ulcer, 2.2 × 3 mm in size involving the anterior 1/3^rd ^of the stroma. Scarring was noted at the peripheral edges of the ulcer. Smears prepared from scrapings obtained from the base and the leading edges of the ulcer were negative but cultures revealed a black *Aspergillus *(designated as strain 138/07). She was advised to continue natamycin eye drops with itraconazole eye ointment. The ulcer healed in two weeks time with complete resolution of the infiltration. During follow-up after 10 months, our patient had a macular grade corneal scar with a best-corrected visual acuity of 6/18.

**Figure 1:**
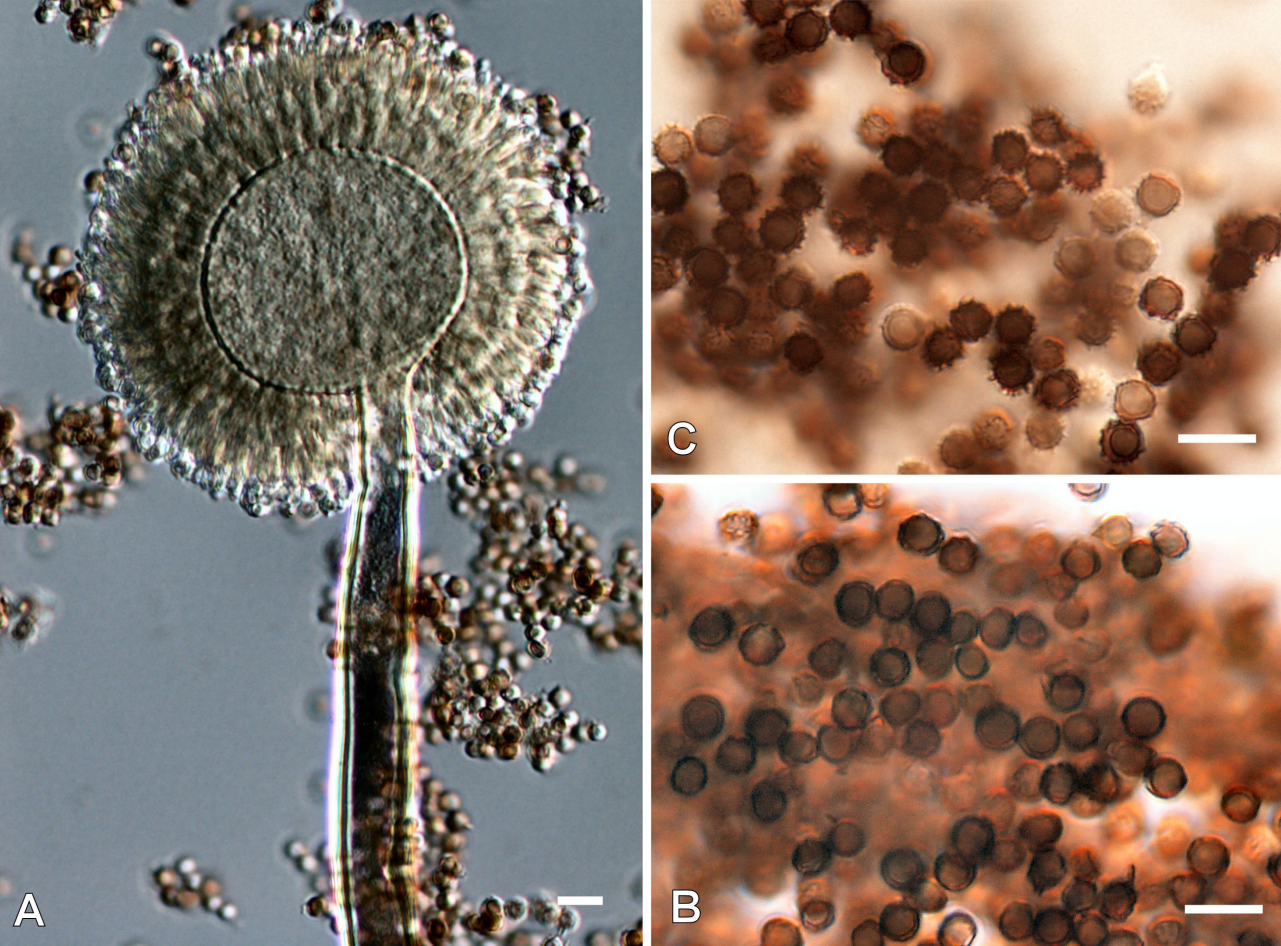
**Micromorphology of *A. brasiliensis***. A: conidiophores, B: conidia of the corneal isolate 138/07; C: conidia of the type strain CBS 101740. Scale bar: 10 μm.

Both isolates were originally suspected to belong to *Aspergillus *section *Nigri *based on macro- and micromorphological characteristics (Figure 1). Colonies were first white then dark brown to black. Exudates were absent, and the reverse of the colony was cream-coloured to light brown. Conidial heads were globose at first and later radiate (Figure 1A), occasionally developing into several conidial columns. Stipes were 700-1700 × 8-13 mm, walls were thick, smooth and pale brown. The vesicles were 30-45 mm wide, nearly globose, biseriate. Metulae were covering virtually the entire surface of the vesicle, measuring 22-30 × 3-6 mm; phialides were flask-shaped, 7-9 × 3-4 mm, conidia subglobose, 3.5-4.8 mm in diameter, echinulate [3]. Interestingly, conidia of the keratitis isolates were not ornamented with tubercules and warts but were smooth walled (Figure 1B), in contrast to the type strain CBS 101740 (Figure 1C). DNA isolation, amplification of a segment of the β-tubulin gene and sequence analysis were carried out as described previously [2]. The partial β-tubulin sequences of strains 832/06 and 138/07 were submitted to the GenBank database under the accession numbers EU600387 and EU600386, respectively. The sequences of the case isolates proved to be completely identical to each other as well as to the corresponding sequence of CBS 101740, the type strain of *A. brasiliensis *[3].

The E-test method (AB BIODISK, Solna, Sweden) for moulds was used to determine the minimal inhibitory concentration (MIC) values of the isolates to amphotericin B, fluconazole, ketoconazole, itraconazole and voriconazole according to the instructions of the manufacturer (Etest technical guide 10). The MIC of natamycin (5% suspension, Sun Pharmaceutical Ind. Ltd., Halol, India), econazole (2% suspension, Aurolab, Madurai, India) and clotrimazole (1% suspension, Aurolab, Madurai, India) were determined by the broth microdilution technique NCCLS M38-A [4].

Table 1 shows the antifungal susceptibility data of the two case isolates. Both of them were resistant to fluconazole (MIC>256 μg/ml), and clotrimazole MIC-values were also higher than 32 μg/ml. Natamycin MICs were similar (1 μg/ml) against these isolates. MICs of other antifungal agents (itraconazole, ketoconazole, voriconazole, econazole, amphotericin B) were 1 μg/ml or lower, but these values were 1 or 2 two-fold dilution-step higher in the case of the isolate 832/06.

**Table 1:** MIC values (μg/ml) of antifungal drugs towards the two A. brasiliensis isolates

	*A. brasiliensis *832/06	*A. brasiliensis *138/07
Itraconazole^a^	1	0.25
Ketoconazole^a^	0.5	0.125
Voriconazole^a^	0.064	0.032
Amphotericin B^a^	0.125	0.064
Econazole^b^	0.032	0.016
Clotrimazole^b^	>32	>32
Fluconazole^a^	>256	>256
Natamycin^b^	1	1

^a^determined by the Etest method

^b^determined by the NCCLS broth microdilution method

Living cultures from case 1 and case 2 were deposited in the Centraalbureau for Schimmelcultures (strain numbers: CBS 122724 and CBS 122723, respectively).

## Discussion

From *Aspergillus *section *Nigri*, only *A. niger *has been reported to date as a possible causative agent of fungal keratitis [1]. In a study from North India, *A. niger *was found to be the most common among the *Aspergillus *species causing keratitis, in 64 out of 78 cases [5]. However, the isolates in this previous study were identified on the basis of their macroscopic and microscopic morphology only, and the identifications were not confirmed by molecular techniques. Black Aspergilli are one of the most difficult groups in classification and identification [6]. Molecular approaches revealed that there is a high biodiversity among them, but that taxa are difficult to be recognized solely on their phenotypic characters [6]. In both cases described in this report, partial sequence analysis of the β-tubulin gene revealed that the isolates belong to the *A. brasiliensis *species. These two *A. brasiliensis *strains were part of six keratitis isolates from *Aspergillus *section *Nigri*, suggesting that this recently described species may be responsible for a significant proportion of corneal infections caused by black *Aspergilli*.

*A. brasiliensis *is a biseriate species closely related to *A. niger *and *A. tubingensis*. This new species is known from soil from Brazil, Australia, USA and the Netherlands, and from grape berries from Portugal, indicating a cosmopolitan distribution [3]. *A. brasiliensis *can be distinguished from other black *Aspergilli *based on intergenic transcribed spacer region, β-tubulin and calmodulin gene sequences, by amplified fragment length polymorphism analysis, by extrolite profiles [3,6] as well as by detecting sequence variations contained in an about 180-bp region of the calmodulin gene with the aid of fluorescence-based SSCP analysis by capillary electrophoresis [7]. Isolates of this species were found to produce naphtho-γ-pyrones, tensidol A and B and pyrophen in common with *A. niger *and *A. tubingensis*, but also several unique compounds, justifying their treatment as representing a separate species [3]. The type strain of the species, *A. brasiliensis *CBS 101740 was also shown to produce xylanase and thermostable beta-xylosidase activities [8].

Although natamycin inhibited the growth of the isolates *in vivo *at low concentration (1 μg/ml), use of this antifungal agent in monotherapy was not successful. This could possibly be due to poor ocular penetration [9]. However, it has been reported that natamycin monotherapy is associated with a poor outcome in *Aspergillus *keratitis [10]. In combination with itraconazole, the treatment was effective in case 2, where the strain was more sensitive for this triazole with a lower MIC value. Other clinical studies mentioned its efficacy in the treatment of corneal ulcers caused by *Aspergillus *spp [9,11]. Case 1 was more complicated: the combined therapy (natamycin plus topical itraconazole and oral ketoconazole) did not resolve the problem. Therapeutic corneal transplantation and administraion of intracameral amphotericin B were needed to eradicate the infection.

## Conclusion

The presented cases indicate that significant differences may occur between the severities of keratitis caused by individual isolates of *A. brasiliensis*. To the best of our knowledge, these cases of fungal keratitis are the first reports on the involvement of *A. brasiliensis *in human infections.

## Consent

Written informed consent was obtained from our patients for publication of this case report and accompanying images. A copy of the written consent is available for review by the Editor-in-Chief of this journal.

## Competing interests

The authors declare that they have no competing interests.

## Authors' contributions

RR, RA, PM, CV, MB, CM, RAS, and LK were involved in the conception and design of the study, while PM, ID, SK, JV, TMN, VN, and LK did the analysis and interpretation.

PM, SK, TMN, LK, JV, ID, and RAS wrote the article, while RR, RA, CV, VN, MB, and CM did the critical revision of the article.

PM, JV, SK, RR, RA, ID, TMN, CV, MB, VN, RAS, CM, and LK had final approval of the article, while PM, RR, RA, ID, SK, JV, TMN, RAS, and LK took charge of the data collection. RR, RA, VN, CV, and RAS provided the materials, patients, and resources.

PM and LK obtained the funding, while PM, LK and JV did the literature search. VN, CV, CM, RAS, and MB provided administrative, technical, or logistic support.

All authors have read and approved the final manuscript.
